# Demographically Calibrated Norms for Two Premorbid Intelligence Measures: The Word Accentuation Test and Pseudo-Words Reading Subtest

**DOI:** 10.3389/fpsyg.2018.01950

**Published:** 2018-10-11

**Authors:** Rocio Del Pino, Javier Peña, Naroa Ibarretxe-Bilbao, David J. Schretlen, Natalia Ojeda

**Affiliations:** ^1^Neurodegenerative Diseases Group, BioCruces Health Research Institute, Barakaldo, Spain; ^2^Department of Methods and Experimental Psychology, University of Deusto, Bilbao, Spain; ^3^Department of Psychiatry and Behavioral Sciences, Johns Hopkins University School of Medicine, Baltimore, MD, United States

**Keywords:** normative data, premorbid intelligence, premorbid IQ, pseudo-words, WAT

## Abstract

The Word Accentuation Test (WAT, Spanish adaptation of the NART) and the Pseudo-Words (PW) Reading subtest from the Battery for Reading Processes Assessment-Revised (PROLEC-R) are measures to estimate premorbid IQ. This study aims to develop demographically calibrated norms for these premorbid measures in a representative sample of the adult Spanish population in terms of age, education, and sex. A sample of 700 healthy participants from 18 to 86 years old completed the WAT and the PW Reading subtest. The effect of age, years of formal education, and sex on WAT total score, PW total score, and time to complete the PW task (PW time) were analyzed. Percentiles and scalar scores were obtained for each raw score according to nine age ranges and individual education levels. The results indicated a significant effect of age and education on the premorbid performance assessed, with no significant effect of sex. Age and education explained from 1.9 to 33.2% of the variance in premorbid IQ variables. Older participants with fewer years of education obtained worse premorbid IQ estimates. This premorbid IQ estimation decline started in the 56–65 age range for WAT total score and PW time, whereas it started in the 71–75 age range for PW total score. This study reports the first demographic-calibrated norms for WAT and PW Reading subtest for Spanish-speaking population. Even though the influence of age and years of education on premorbid IQ measures was confirmed, the PW Reading subtest showed to be more resistant to decline in elderly population than the WAT.

## Introduction

The neuropsychological performance assessment requires knowledge of the previous intellectual level of the person being assessed (Lezak, 2004; Alves et al., 2013). The detection of neuropsychological deficits rests upon the comparison of an individual’s current cognitive functioning with an estimate of his/her premorbid IQ (Crawford, 1992; Law and O’Carroll, 1998; McGurn et al., 2004). Estimation of premorbid function is becoming increasingly recognized as a crucial component of neuropsychological assessment in both research and clinical practice due to its relevance in the diagnosis and rehabilitation process (Law and O’Carroll, 1998; Lowe and Rogers, 2011; Vakil, 2012). It helps clinicians and researchers to accurately diagnose the level of cognitive decline in different pathologies such as schizophrenia (Gomar et al., 2011; Khandaker et al., 2011), traumatic brain injury (Freeman et al., 2001; Kesler et al., 2003), dementia (Law and O’Carroll, 1998; McFarlane et al., 2006; Hessler et al., 2013), or normal aging (Sierra et al., 2010; Lowe and Rogers, 2011). However, there is a lack of normative data on premorbid IQ measures, specifically for Spanish population. Thus, neuropsychologists have to use normative data from other populations, mainly from the Unites States or South America. Nevertheless, several studies showed that age, education, language, and culture have a significant impact on neuropsychological performance (Ardila, 2007; del Pino et al., 2015).

Regarding premorbid IQ instruments, the most common approaches for estimating premorbid IQ are: (a) demographic-based regression equations (Barona et al., 1984); (b) cognitive instruments that measure reading ability (i.e., the National Adult Reading Test-NART, Nelson, 1982), or assessing patterns of performance (i.e., the Vocabulary subtest from Wechsler Adult Intelligence Scale-Revised: WAIS-R, Wechsler, 1981); and (c) the combination of demographic-based regression equations with cognitive instruments such as reading tests (Crawford et al., 1989a).

Demographic-based regression equations which include demographic variables (i.e., age, level of formal education, or social class) are known to be related to scores on intelligence tests, and could yield information on an individual’s premorbid level of intellectual functioning (Barona et al., 1984; Crawford, 1992; Bright et al., 2002). This method uses demographic data in a multiple regression equation to determine the index of premorbid IQ (Eppinger et al., 1987; Crawford et al., 1989b). One of the most widely used is the Barona index (Barona et al., 1984) which is a demographically based regression equation to estimate premorbid IQ using the WAIS-R (Wechsler, 1981). This formula displays adequate predictive validity in estimating WAIS-R Full Scale IQ (FSIQ) for individuals in the borderline and average intelligence ranges (Barona et al., 1984). Nonetheless, the Barona index may provide significant under- or overestimates in cases where premorbid IQ is above 120 or below 69 (Barona et al., 1984; Franzen et al., 1997; Griffin et al., 2002).

Another effective procedure for estimating premorbid ability relies upon cognitive instruments which measure an over learned skill such as reading or assess patterns of performance (Nelson and McKenna, 1975; Del Ser et al., 1997; Russell et al., 2000; Contador et al., 2015). Several authors have criticized the use of performance tests, such as the vocabulary subtest from the WAIS-R, to estimate the premorbid IQ; because vocabulary is known to decline with aging and is sensitive to brain damage, specifically in the neuroanatomical basis of language (Martin and Fedio, 1983; Del Ser et al., 1997; Sierra et al., 2010; Hebben et al., 2011; de Oliveira et al., 2014). However, reading ability becomes, with practice, an automatic ability that is highly resistant to cognitive impairment (Del Ser et al., 1997; Russell et al., 2000; Khandaker et al., 2011; Harman-Smith et al., 2013; Hessler et al., 2013). Hence, instruments based on reading irregular words (i.e., NART) (Nelson, 1982) or based on pseudo-words (PW) (i.e., Spot-the-Word test) (Baddeley et al., 1993) are used to estimate premorbid IQ.

More recent approaches employ multiple regression equations to predict cognitive performance using both demographic variables and cognitive instruments as predictors (Griffin et al., 2002; Harnett et al., 2004; Ball et al., 2007; Sierra Sanjurjo et al., 2015). For example, the Oklahoma Premorbid Intelligence Estimation (OPIE) estimates premorbid IQ by combining demographic variables (age, education, occupation, and race) with current performance on the WAIS-R (Vocabulary and Picture Completion subtests) (Scott et al., 1997; Griffin et al., 2002). The OPIE showed a wider range of premorbid IQ scores in which no systematic under- or over-estimation was performed compared to other available techniques (Griffin et al., 2002). However, several authors reported incremental accuracy when the NART or an oral reading test is combined with demographic information instead of using the WAIS-R; which makes this last procedure the most appropriate one (Crawford et al., 1990; Griffin et al., 2002; Harnett et al., 2004; Lezak, 2004; Ball et al., 2007; Sierra Sanjurjo et al., 2015). Therefore, it seems to be important to have normative data of oral reading test to estimate accurately the premorbid IQ.

The most popular reading test to estimate premorbid IQ is the NART which was created for English speakers. It was adapted to different languages, but it seems impossible to build a Spanish model due to the peculiarities of the language since every Spanish word is read in a regular way. English is an opaque language, with highly irregular rules for pronunciation while Spanish is a transparent language in which any written word or non-word can be read aloud generating the sounds from letters. Although Spanish accentuation is quite regular, the words that do not follow accentuation rules must be ortho-graphically stressed. However, there are exceptions; and in order to read correctly the words, the reader needs to see the written accentuation mark or have previous knowledge of the correct accentuation of the word when this mark is not present. As a result, Del Ser et al. (1997) designed the Word Accentuation Test (WAT), the Spanish adaptation of the NART, to estimate the premorbid IQ in dementia (Del Ser et al., 1997). This adaptation is based on the prosodic accentuation of infrequent Spanish words and its use has been expanded to measure premorbid functioning in patients with schizophrenia (Gomar et al., 2011), cognitive impairment and dysexecutive syndrome (Sierra et al., 2010), as well as healthy people without cognitive impairment (Sierra et al., 2010; Contador et al., 2015). Nevertheless, there is currently no normative data available for this instrument. Concerning reading PW tests, the Spot-the-Word test (Baddeley et al., 1993) was proposed as an adequate instrument to assess premorbid IQ in older adults with normal aging as well as in patients with dementia (Friedman et al., 1992; Patterson et al., 1994; McFarlane et al., 2006). However, to our knowledge, there is no Spanish version of this test. A similar test is available for the Spanish population as part of a wider battery for assessing reading processes (PROLEC-R) (Cuetos et al., 2007). Nonetheless, it was initially created for children and no norms are available for adults.

Within this framework, in order to obtain accurate and calibrated norms for potential Spanish premorbid IQ instruments, this study seeks to normalize and standardize the WAT and the PW Reading subtest from PROLEC-R in the Spanish adult population. Moreover, we also aim to analyze the relationship between these two premorbid IQ measures to explore their potential equivalency or specific characterization in the healthy population. It is expected to provide alternative methods to accurately estimate premorbid IQ which could enrich clinicians’ and researchers’ decisions based on the specifications of the population they work with.

## Materials and Methods

### Participants

Participants were recruited by “word of mouth” from eight different geographical locations in Spain. The final sample size was seven hundred healthy participants (age ranged from 18 to 86 years old) who were selected by taking into account the sociodemographic characteristics of the Spanish population (National Institute of Statistics [NIS], 2012) data. Inclusion criteria were as follow: (1) healthy subjects, without previous history of physical or mental illness that significantly compromises the individual’s intellectual or cognitive functioning; (2) age ranging from 18 to 90 years old; (3) Spanish mother tongue or bilingual; (4) Spanish population representative of different age ranges and educational levels (National Institute of Statistics [NIS], 2012); (5) voluntary participation; (6) signed informed consent; and (7) ≥score of 26 in the Telephone Interview for Cognitive Status (TICS) (Brandt et al., 1988). Exclusion criteria were: (1) medical history of physical or mental illness that affect their cognitive performance; (2) severe cognitive impairment; or (3) sensory limitations (visual or auditory) which cannot be adequately compensated by corrections (glasses or hearing aids).

The project was approved by the Ethics Committee at the University of Deusto, Bilbao, Spain. All subjects were volunteers and provided written informed consent prior to their participation in the study, in accordance with the Declaration of Helsinki.

### Measures

The WAT assesses Spanish speakers’ premorbid IQ by reading the correct pronunciation of 30 low frequency Spanish words whose accents have been removed (González Montalvo, 1991; Del Ser et al., 1997). In this test examinees must demonstrate their knowledge of the correct accentuation of each word. Spanish words can be classified into one of four groups depending on the position of their stress. When last syllable is stressed, the word belongs to the “*aguda*” category. “*Aguda*” words need written accent when they end in a vowel or the consonants “-n” or “-s.” If the stress falls on the second to last syllable, it is classified as a “*llana*.” “*Llanas*” form the majority of the Spanish lexicon and most of them end in a vowel or the consonants “-n” or “-s.” Written accent is carried only by those “*llanas*” ending in a consonant different from “-n” or “-s” preceded by a vowel or ending in any two consecutive consonants. If the stress is placed on the third last or the fourth to last syllable, they are categorized as “*esdrújulas*” or “*sobresdrújulas*,” respectively. In either of the last two categories, the stressed syllable must have written accent. This knowledge cannot be inferred unless they have been previously exposed to the word. This experience is routinely acquired through education and language experience. The Spanish instructions are presented in the **Supplementary Material** (**Supplementary Figure 1**). The person is asked to read each word aloud. The words are printed on white paper in bold black capital letters (see **Supplementary Figure 2**). The total score is the number of words correctly read (from 0 to 30). The test is administered individually and takes 2–3 min.

The PW Reading subtest from the PROLEC-R (Cuetos et al., 2007) assesses the sub-lexical processes (phonological path) and reading fluency. The person is asked to read aloud each PW as quickly as possible. Time to complete the task and accuracy of reading PW are recorded and scored. Stimuli include 40 non-existent words (PW). Like the WAT, the total score is the number of words correctly read (from 0 to 40) and the test takes 3–4 min.

### Data Analysis

Data were analyzed using the Statistical Package for the Social Science (SPSS), version 20. Sociodemographic and cognitive variables were analyzed with a Student’s *t*-test and a chi-squared test was used for categorical variables.

The relationship between the cognitive performance in each of the measures included (WAT total score, PW total score, and time to complete the task in PW) and the sociodemographic characteristics such as age, education, and sex were analyzed by linear regression. Coefficients of correlation (r) and determination (r^2^) of raw scores were determined.

Age was codified in nine ranges (18–25, 26–35, 36–45, 46–55, 56–65, 66–70, 71–75, 76–80, 81–86). The differences in cognitive performance according to these age ranges were analyzed by one-way ANOVA and *post hoc* Tukey’s HSD test was also analyzed.

The normative data and the standardization for the WAT total score, PW total score, and time to complete the task in PW were obtained. The normative procedure carried out was the following: (1) To ensure a normal distribution, the raw scores were assigned their corresponding percentiles (Pc) by the frequency distribution and Pc were transformed into scalar scores (SS) (from 2 to 18) for each age range (Ivnik et al., 1992; Peña-Casanova et al., 2009, 2012; Testa et al., 2009; Smerbeck et al., 2012; del Pino et al., 2015); (2) This transformation of raw scores to SS adjusted by age (SSa) produced a normalized distribution; and (3) Multiple linear regressions were analyzed in order to create the final normative data adjusted by years of education. The following equation was used (Peña-Casanova et al., 2009, 2012; del Pino et al., 2015):

(1) SSn (normalized)=SSa−(β*[Years of education−12])

## Results

The sociodemographic characteristics of the total sample are shown in **Table 1**. The sample was composed of 305 males (mean age = 45.5, SD = 18.0; mean years of education = 13.1, SD = 5.1) and 395 females (mean age = 44.6, SD = 17.9; mean years of education = 12.7, SD = 4.8). No significant differences were found for age or years of formal education between males and females (age: *t* = 0.61; *p* = 0.54; years of education: *t* = 0.86; *p* = 0.39). However, there were statistical differences in employment status (χ^2^ = 48.68; *p* < 0.001) and in professional/working class (χ^2^ = 27.34; *p* < 0.001) in the total sample assessed. 50.4% of the sample were working at the time of the assessment; 27.3% of the sample were classified as high technical in the professional/working class while 30.7% of the sample were unskilled.

**Table 1:** Sociodemographic characteristics of the sample.

	Males (*n* = 305)		Females (*n* = 395)		Total (*N* = 700)		
	*n*	%	*n*	%	*n*	% Total	χ^2^ (*p*)
**Age range**							9.5 (0.30)
18–25	42	13.8	78	19.7	120	17.1	
26–35	73	23.9	76	19.2	149	21.3	
36–45	42	13.8	51	12.9	93	13.3	
46–55	57	18.7	74	18.7	131	18.7	
56–65	42	13.8	61	15.4	103	14.7	
66–70	17	5.6	16	4.1	33	4.7	
71–75	9	3.0	18	4.6	23	3.9	
76–80	13	4.3	10	2.5	23	3.3	
81–86	10	3.3	11	2.8	21	3.0	
**Educational level**							0.2 (0.98)
0–6	39	12.8	54	13.7	93	13.3	
7–10	52	17.0	70	17.7	122	17.4	
11–12	47	15.4	59	14.9	106	15.1	
12	167	54.8	212	53.7	379	54.1	
**Marital status**							4.7 (0.19)
Single	117	38.4	156	39.5	273	39.0	
Married	163	53.4	188	47.6	351	50.1	
Divorced/separated	11	3.6	23	5.8	34	4.9	
Widowed	14	4.6	28	7.1	42	6.0	
**Employment status**							48.6 (<0.001)
Unemployed	28	9.2	29	7.3	57	8.1	
Student	36	11.8	67	17.0	103	14.7	
Homemaker	3	1.0	58	14.7	61	8.7	
Retired	66	21.6	60	15.2	126	18.0	
Active	172	56.4	181	45.8	353	50.4	
**Professional/working class**							27.3 (<0.001)
Unskilled	83	27.2	132	33.4	215	30.7	
Skilled	55	18.0	47	11.9	102	14.6	
Administrative	9	3.0	43	10.9	52	7.4	
Middle technical professional	37	12.1	43	10.9	80	11.4	
High technical professional	100	32.8	91	23.0	191	27.3	

The mean and standard deviations of the premorbid performance in the sample was 24.1 (SD = 4.9) for WAT total score, 36.9 (SD = 3.8) for PW total score, and 49.0 (SD = 23.2) seconds to complete the PW task. The measures were highly correlated with one another. The WAT total score correlated positively with the PW total score (*r* = 0.50; *p* < 0.001) and negatively with time in PW (*r* = −0.55; *p* < 0.001). Those results suggested that both measures (WAT and PW) evaluate similar constructs. Hence, a composite variable of WAT and PW, named premorbid intelligence (PI) composite score, was created in order to analyze the relationship between the sociodemographic characteristics and this construct. Coefficients of correlation (r) and determination (r2) are shown in **Table 2**. There was a significant effect of age and years of education on performance in WAT total score, in PW total score, in PW time, and in PI composite score. Nevertheless, sex was not significant. The percentage of variance explained by age and education ranged from 1.9 to 9.2% and 20.5 to 33.2%, respectively.

**Table 2:** Correlation analysis between WAT total score, PW total score, time to complete the task in PW (subtest from PROLEC-R), PI composite score and demographic characteristics.

Cognitive domains	Coefficients of correlation and determination			
	Age		Years of education	
	*r*	*r*^2^	*r*	*r*^2^
WAT	−0.30^∗∗∗^	0.09	0.56^∗∗∗^	0.31
PW	−0.41^∗∗∗^	0.05	0.45^∗∗∗^	0.20
PW time	0.53^∗∗∗^	0.08	−0.57^∗∗∗^	0.32
PI composite score	−0.41^∗∗∗^	0.02	0.58^∗∗∗^	0.33

*^∗∗∗^<0.001; WAT, Word Accentuation Test total score; PW, Total Score of Pseudo-words Reading subtest from PROLEC-R; PW time, time to complete the PW task in seconds; PI composite score, Premorbid Intelligence (composite variable from WAT and PW).*

**FIGURE 1:**
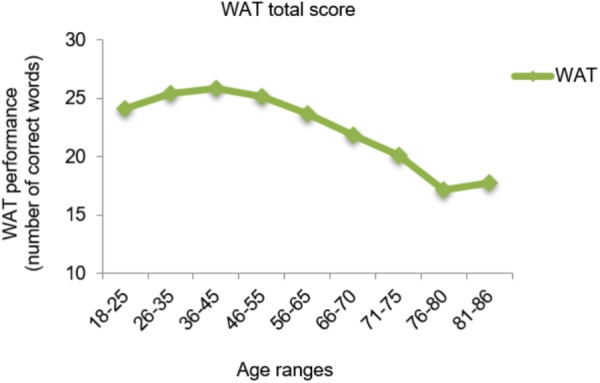
Cognitive performance of the WAT total score by age ranges.

**FIGURE 2:**
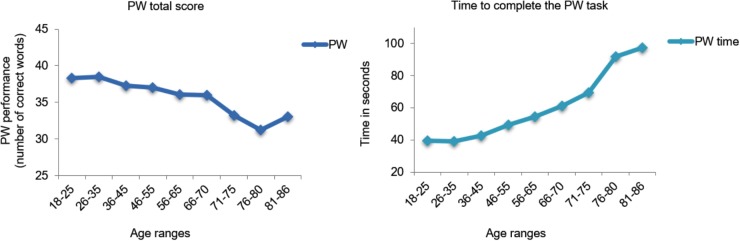
Cognitive performance of the PW Reading subtest (total scores) and time to complete the PW task in seconds by age ranges.

As shown in **Figures 1**, **2**, the premorbid performance is presented graphically according to nine age ranges for the WAT total score [*F*(8,691) = 20.7; *p* < 0.001], the PW total score [*F*(8,688) = 22.1; *p* < 0.001], and time to complete the task in PW [*F*(8,688) = 49.2; *p* < 0.001]. A general pattern is observed in all the variables analyzed by *post hoc* Tukey’s HSD test; older people obtained worse cognitive performance than young people. Specifically, the performance started to decline in the 56–65 age range for WAT total score (*p* < 0.001) and time to complete the task in PW (*p* < 0.001), while the PW total score declined after 71 years of age (*p* < 0.001).

**Tables 3**, **4** display the first step to obtain the SS adjusted by age (SSa) and the Pc of their corresponding raw scores. The second step to obtain the normalized SS (SSn), adjusted by age and years of education for the WAT total score, PW total score and time to complete the task in PW is shown in **Tables 5**, **6**. These two tables show the years of education in the upper right and the SSa and the Pc obtained in the previous tables (see **Tables 3**, **4** according to the age of the participant) are in the two left columns. As an example, the interpretation procedure for a 46 year-old participant with 8 years of education that obtained a score of 26 in the WAT is the following: Step 1, **Table 3** shows his/her corresponding SSa and Pc (SSa = 10; Pc = 44–56); Step 2, check **Table 5** in order to obtain SSn. Years of education (8 years) appears in the upper right and the SSa and the Pc obtained previously (SSa = 10; Pc = 44–56) are now up to a SSn of 12 and a Pc of 69–79. As a result, his/her actual SSn is above the mean.

**Table 3:** Scalar scores, percentiles and raw scores for the entire sample and according to age ranges (from 18 to 55 years old).

Step 1 (18–55 years old)					Raw scores											
SS_a_	Pc	Entire sample			18–25			26–35			36–45			46–55		
	
		WAT	PW	PW time	WAT	PW	PW time	WAT	PW	PW time	WAT	PW	PW time	WAT	PW	PW time
2	<1	0–5	0–17	214–240	0–13	0–26	65	0–11	0–33	57–67	0–15	0–29	75–90	0–9	0–24	84–89
3	1	6–9	18–25	125–213	14	27–32	–	12–13	34	55–56	16	–	–	10–13	25	–
4	2–3	10–13	26–29	96–124	15–18	33	57–64	14–17	35	52–54	17–18	30–31	70–74	14–17	26–30	77–83
5	4–6	14–15	30–31	79–95	19	34–35	55–6	18–20	36	49–51	19–20	32	60–69	18–19	31–32	75–76
6	7–12	16–18	32–34	67–78	20	36	51–54	21–22	37	46–48	21–22	33–35	56–59	20–21	33–34	64–74
7	13–20	19–21	35	59–66	21	37	47–50	23	38	44–45	23	–	53–55	22–23	35	59–63
8	21–30	22–23	36–37	52–58	22–23	38	44–46	24	–	40–43	24–25	36–37	48–52	24	36	54–58
9	31–43	24–25	38	47–51	24	39	41–43	25	39	38–39	26	–	44–47	25	37	50–53
10	44–56	26	–	43–46	25	–	38–40	26	–	36–37	27	38	40–43	26	38	46–49
11	57–68	27	39	39–42	26	–	36–37	27	–	33–35	28	–	37–39	27–28	39	40–45
12	69–79	28	–	36–38	27	–	33–35	28	–	31–32	–	39	33–36	–	–	37–39
13	80–86	29	–	33–35	–	–	30–32	29	–	30	29	–	31–32	–	–	34–36
14	87–92	–	–	30–32	28	–	28–29	–	–	28–29	–	–	29–30	29	–	31–33
15	93–95	–	–	27–29	29	–	26–27	–	–	26–27	–	–	27–28	–	–	28–30
16	96–97	–	–	25–26	–	–	24–25	–	–	23–25	–	–	25–26	–	–	26–27
17	98	–	–	22–24	–	–	22–23	–	–	22	–	–	24	–	–	25
18	>99	30	40	0–21	30	40	0–21	30	40	0–21	30	40	0–23	30	40	0–24

*SS_a_, Scalar scores adjusted by age; Pc, percentiles; WAT, Word Accentuation Test total score; PW, Total Score of Pseudo-words Reading subtest from PROLEC-R; PW time, time to complete the PW task in seconds. Interpretation example: a 46 year-old participant with 8 years of education obtained a score of 26 in the WAT; Step 1: **Table 3** shows that his/her corresponding SS_a_ and Pc is 10 and 44–56, respectively. Step 2: check **Table 5** for the SS_n_.*

**Table 4:** Scalar scores, percentiles and raw scores according to age ranges (from 56 to 86 years old).

Step 1 (56–86 years old)			Raw scores													
SS_a_	Pc	56–65			66–70			71–75			76–80			81–86		
	
		WAT	PW	PW time	WAT	PW	PW time	WAT	PW	PW time	WAT	PW	PW time	WAT	PW	PW time
2	<1	0–8	0–25	118–124	0–8	0–25	118–133	0–1	0–5	141–144	0–1	0–13	218–240	0–4	0–15	195–240
3	1	–	–	–	–	–	–	–	–	–	–	–	–	–	–	–
4	2–3	9	–	–	–	–	–	–	–	–	–	–	–	–	–	–
5	4–6	10–11	26–27	86–117	9–11	26–27	86–117	2–4	6–23	102–140	2–3	14–16	–	–	16–19	–
6	7–12	12–13	28–29	81–85	12–13	28–29	81–85	5–13	24–30	94–101	4–10	17–23	181–217	5	20–25	154–194
7	13–20	14–16	30–33	71–80	14–16	30–33	71–80	14–15	31	84–93	11	24	137–180	–	26–31	136–153
8	21–30	17–18	34–35	68–70	17–18	34–35	68–70	16–17	32	80–83	12–14	25–30	87–136	6–10	32	110–135
9	31–43	19–21	36	63–67	19–21	36	63–67	18–19	33–34	73–79	15–17	31	80–86	11–13	33	98–109
10	44–56	22–24	37–38	57–62	22–24	37–38	57–62	20–21	35	64–72	18	32–34	68–79	14–15	34	72–97
11	57–68	25–26	–	54–56	25–26	–	54–56	22–25	–	61–63	19–21	35	62–67	16–19	35–36	66–71
12	69–79	27	–	45–53	27	39	45–53	26	36–38	51–60	22–23	36–37	54–61	20–24	37	59–65
13	80–86	28–29	39	41–44	28	–	41–44	27–28	–	42–50	24–25	38	41–53	25–26	38	55–58
14	87–92	–	–	36–40	29	–	36–40	29	39	37–41	26–29	–	24–40	27–29	–	49–54
15	93–95	–	–	33–35	–	–	33–35	–	–	12–36	–	–	12–23	–	–	–
16	96–97	–	–	–	–	–	–	–	–	–	–	–	–	–	–	–
17	98	–	–	–	–	–	–	–	–	–	–	–	–	–	–	–
18	>99	30	40	0–32	30	40	0–32	30	40	0–11	30	39–40	0–11	30	39–40	0–48

*SS_a_, Scalar scores adjusted by age; Pc, percentiles; WAT, Word Accentuation Test total score; PW, Total Score of Pseudo-words Reading subtest from PROLEC-R; PW time, time to complete the PW task in seconds.*

**Table 5:** Scalar scores normalized from WAT total score adjusted by age and education.

Step 2																						
WAT		Years of education																				
SS_a_	Pc	0	1	2	3	4	5	6	7	8	9	10	11	12	13	14	15	16	17	18	19	20
2	<1	9	8	7	7	6	6	5	5	4	4	3	3	2	1	1	0	0	−1	−1	−2	−2
3	1	10	9	8	8	7	7	6	6	5	5	4	4	3	2	2	1	1	0	0	−1	−1
4	2–3	11	10	9	9	8	8	7	7	6	6	5	5	4	3	3	2	2	1	1	0	0
5	4–6	12	11	10	10	9	9	8	8	7	7	6	6	5	4	4	3	3	2	2	1	1
6	7–12	13	12	11	11	10	10	9	9	8	8	7	7	6	5	5	4	4	3	3	2	2
7	13–20	14	13	12	12	11	11	10	10	9	9	8	8	7	6	6	5	5	4	4	3	3
8	21–30	15	14	13	13	12	12	11	11	10	10	9	9	8	7	7	6	6	5	5	4	4
9	31–43	16	15	14	14	13	13	12	12	11	11	10	10	9	8	8	7	7	6	6	5	5
10	44–56	17	16	15	15	14	14	13	13	12	12	11	11	10	9	9	8	8	7	7	6	6
11	57–68	18	17	16	16	15	15	14	14	13	13	12	12	11	10	10	9	9	8	8	7	7
12	69–79	19	18	17	17	16	16	15	15	14	14	13	13	12	11	11	10	10	9	9	8	8
13	80–86	20	19	18	18	17	17	16	16	15	15	14	14	13	12	12	11	11	10	10	9	9
14	87–92	21	20	19	19	18	18	17	17	16	16	15	15	14	13	13	12	12	11	11	10	10
15	93–95	22	21	20	20	19	19	18	18	17	17	16	16	15	14	14	13	13	12	12	11	11
16	96–97	23	22	21	21	20	20	19	19	18	18	17	17	16	15	15	14	14	13	13	12	12
17	98	24	23	22	22	21	21	20	20	19	19	18	18	17	16	16	15	15	14	14	13	13
18	> 99	25	24	23	23	22	22	21	21	20	20	19	19	18	17	17	16	16	15	15	14	14

*SS_a_, Scalar scores adjusted by age; WAT, Word Accentuation Test total score. Interpretation example: a 46 year-old participant with 8 years of education obtained a score of 26 in the WAT; Step 1: **Table 3** shows that his/her corresponding SS_a_ and Pc is 10 and 44–56, respectively; Step 2: **Table 5** shows the SS_n_. Years of education (8 years) appears in the upper right and the SS_a_ and the Pc obtained previously (SS_a_ = 10; Pc = 44–56) are now up to an SS_n_ of 12 and a Pc of 69–79.*

**Table 6:** Scalar scores normalized from PW total scores and time to complete the PW task adjusted by age and education.

Step 2 PW																						
	
PW		Years of education																				
SS_a_	Pc	0	1	2	3	4	5	6	7	8	9	10	11	12	13	14	15	16	17	18	19	20
2	<1	6	6	5	5	5	4	4	4	3	3	3	2	2	2	1	1	1	0	0	0	−1
3	1	7	7	6	6	6	5	5	5	4	4	4	3	3	3	2	2	2	1	1	1	0
4	2–3	8	8	7	7	7	6	6	6	5	5	5	4	4	4	3	3	3	2	2	2	1
5	4–6	9	9	8	8	8	7	7	7	6	6	6	5	5	5	4	4	4	3	3	3	2
6	7–12	10	10	9	9	9	8	8	8	7	7	7	6	6	6	5	5	5	4	4	4	3
7	13–20	11	11	10	10	10	9	9	9	8	8	8	7	7	7	6	6	6	5	5	5	4
8	21–30	12	12	11	11	11	10	10	10	9	9	9	8	8	8	7	7	7	6	6	6	5
9	31–43	13	13	12	12	12	11	11	11	10	10	10	9	9	9	8	8	8	7	7	7	6
10	44–56	14	14	13	13	13	12	12	12	11	11	11	10	10	10	9	9	9	8	8	8	7
11	57–68	15	15	14	14	14	13	13	13	12	12	12	11	11	11	10	10	10	9	9	9	8
12	69–79	16	16	15	15	15	14	14	14	13	13	13	12	12	12	11	11	11	10	10	10	9
13	80–86	17	17	16	16	16	15	15	15	14	14	14	13	13	13	12	12	12	11	11	11	10
14	87–92	18	18	17	17	17	16	16	16	15	15	15	14	14	14	13	13	13	12	12	12	11
15	93–95	19	19	18	18	18	17	17	17	16	16	16	15	15	15	14	14	14	13	13	13	12
16	96–97	20	20	19	19	19	18	18	18	17	17	17	16	16	16	15	15	15	14	14	14	13
17	98	21	21	20	20	20	19	19	19	18	18	18	17	17	17	16	16	16	15	15	15	14
18	>99	22	22	21	21	21	20	20	20	19	19	19	18	18	18	17	17	17	16	16	16	15

**Step 2**																						
	
**PW time**		**Years of education**																				

SS_a_	Pc	0	1	2	3	4	5	6	7	8	9	10	11	12	13	14	15	16	17	18	19	20
2	<1	−3	−2	−2	−2	−1	−1	0	0	0	1	1	2	2	2	3	3	4	4	4	5	5
3	1	−2	−1	−1	−1	0	0	1	1	1	2	2	3	3	3	4	4	5	5	5	6	6
4	2–3	−1	0	0	0	1	1	2	2	2	3	3	4	4	4	5	5	6	6	6	7	7
5	4–6	0	1	1	1	2	2	3	3	3	4	4	5	5	5	6	6	7	7	7	8	8
6	7–12	1	2	2	2	3	3	4	4	4	5	5	6	6	6	7	7	8	8	8	9	9
7	13–20	2	3	3	3	4	4	5	5	5	6	6	7	7	7	8	8	9	9	9	10	10
8	21–30	3	4	4	4	5	5	6	6	6	7	7	8	8	8	9	9	10	10	10	11	11
9	31–43	4	5	5	5	6	6	7	7	7	8	8	9	9	9	10	10	11	11	11	12	12
10	44–56	5	6	6	6	7	7	8	8	8	9	9	10	10	10	11	11	12	12	12	13	13
11	57–68	6	7	7	7	8	8	9	9	9	10	10	11	11	11	12	12	13	13	13	14	14
12	69–79	7	8	8	8	9	9	10	10	10	11	11	12	12	12	13	13	14	14	14	15	15
13	80–86	8	9	9	9	10	10	11	11	11	12	12	13	13	13	14	14	15	15	15	16	16
14	87–92	9	10	10	10	11	11	12	12	12	13	13	14	14	14	15	15	16	16	16	17	17
15	93–95	10	11	11	11	12	12	13	13	13	14	14	15	15	15	16	16	17	17	17	18	18
16	96–97	11	12	12	12	13	13	14	14	14	15	15	16	16	16	17	17	18	18	18	19	19
17	98	12	13	13	13	14	14	15	15	15	16	16	17	17	17	18	18	19	19	19	20	20
18	>99	13	14	14	14	15	15	16	16	16	17	17	18	18	18	19	19	20	20	20	21	21

*SS_a_, Scalar scores adjusted by age; PW, Total Score of Pseudo-words Reading subtest from PROLEC-R; PW time, time to complete the PW task in seconds.*

## Discussion

This study provides the first demographically calibrated norms for two premorbid IQ measures with a representative sample of the Spanish population (National Institute of Statistics [NIS], 2012). The normative data for the WAT and the PW Reading subtest from the PROLEC-R are presented according to nine age ranges and individual levels of formal education.

Our results showed that demographic variables such as age and years of formal education have an impact on the premorbid performance (Del Ser et al., 1997; Harnett et al., 2004; Testa et al., 2009; Schretlen et al., 2009; Lowe and Rogers, 2011). In our study, education was a better predictor of these premorbid measures than age. Indeed, years of education explained 33.2% of the variance in the premorbid composite variable (WAT and PW total score), while age only explained 9.0% of variance in the WAT total score. Similarly, Lowe and Rogers (2011) found that the American version of the NART correlated significantly with education, but not with age. In fact, according to the cognitive reserve hypothesis, some authors found that highly educated individuals may also continue to benefit from cognitive reserve even after the diagnosis of dementia, showing slower decline in some areas of cognition (Stern et al., 1994; Le Carret et al., 2005).

This study supported the hypothesis that combining demographic variables with reading ability tests provides higher reliable estimates of premorbid IQ (Crawford et al., 1990; Ball et al., 2007; Mathias et al., 2007; Schretlen et al., 2009). Demographic variables are predictors of cognitive performance, and therefore influence the diagnosis of cognitive impairment (Graves et al., 1999; Lezak, 2004). As the results showed, those participants who had completed more years of education had a higher level of premorbid functioning (Lowe and Rogers, 2011), however, premorbid IQ decreased with aging. Reading performance decreased in the 56–65 age range in WAT while the performance in PW started declining in the 71–75 age range. This pointed out that the PW Reading subtest was less influenced by aging than WAT. This indicates that the PW Reading subtest seems to be more resistant to decline with aging, and consequently, it could be a better measure to estimate premorbid IQ in elderly people (Friedman et al., 1992; Patterson et al., 1994). This could be explained by the sub-lexical pathway since PW reading requires the sub-lexical pathway which is more basic than the lexical pathway, and therefore more resistant to decline (Cuetos et al., 2007). In accordance with our results, Patterson et al. (1994) found that reading PW estimates the premorbid IQ more accurately than reading infrequent words. Their results showed larger differences between healthy controls and patients with Alzheimer’s Disease for words read correctly on the NART than in the non-word reading test (PW).

This study might have a direct impact on the clinical field as the normative data provide clinicians and researchers the possibility to estimate the premorbid IQ of patients more accurately, thus making more precise diagnosis. This could reduce the misdiagnosis of neurodegenerative diseases or mild cognitive impairment in elderly people. Furthermore, this study highlights the need that each country should create and follow their own demographically adapted norms taking into account their cultural aspects. According to several authors, United States norms are clearly not appropriate for every country since culture, ethnicity, education, and language among others, influence neuropsychological performance (Buré-Reyes et al., 2013; Ojeda et al., 2014). Hence, norms derived from other cultures are not appropriate to make individual-level diagnoses. Moreover, these findings also emphasize the relevance of calibrating performance according to intraindividual demographics in each country.

Despite the significant contribution to the clinical and scientific field, reporting normative data with a large representative sample of Spanish population, this study has several limitations. One common limitation to all normative studies is that normative data are limited to the characteristics of those tested (Mitrushina, 2005; Peña-Casanova et al., 2009; del Pino et al., 2015). However, this study tried to assess a wide variety of adult Spanish people with all kind of possible sociodemographic characteristics (National Institute of Statistics [NIS], 2012). Even though a very careful procedure was followed in the stratification and recruitment process, it was not possible to perform it by regions in Spain. Consequently, the population from the Basque Country region was overrepresented. For future studies, it would be interesting to include the representative percentage of people from different areas around Spain according to the NIS data and to increase the number of elderly people in the sample. Including illiterate people in the sample could be another limitation. Education was not compulsory in Spain until the General Education Act was passed in 1970. Compulsory education was from 6 to 14 years of age, but it was extended to the age of 16 in 1994. Accordingly, illiteracy is not common in Spain. Nonetheless, people with only 2 or 3 years of education were also included in the study.

## Conclusion

In conclusion, this study reports the first normative data for the WAT and PW Reading subtest from PROLEC-R adapted by age and years of formal education of the Spanish population in order to estimate accurately premorbid IQ. Additionally, to our knowledge, this is the first study that uses the PW Reading subtest for adults. Future research is planned to estimate the validity of these two measures estimating premorbid IQ among the clinical population.

## Author Contributions

All authors listed have made substantial, direct and intellectual contributions to the work, and approved it for publication. NO and DS came up with the idea for this project. NO, JP, and RP contributed to the project and managed all steps of the research process. RP performed the data collection, data entry, data analysis, and manuscript writing. The co-authors contributed equally to this research in the areas of research design, and revision of the manuscript writing.

## Conflict of Interest Statement

The authors declare that the research was conducted in the absence of any commercial or financial relationships that could be construed as a potential conflict of interest.

## Supplementary Material

FIGURE S1 Instructions and correction of the WAT in Spanish for the rater’s use.

FIGURE S2 Stimulus sheet of the WAT.
