# Supplementary figures and images for: Demographically Calibrated Norms for Two Premorbid Intelligence Measures: The Word Accentuation Test and Pseudo-Words Reading Subtest
Source: Front Psychol. 2018 Oct 11;9:1950. doi: 10.3389/fpsyg.2018.01950 (PMC6193077; doi:10.3389/fpsyg.2018.01950)

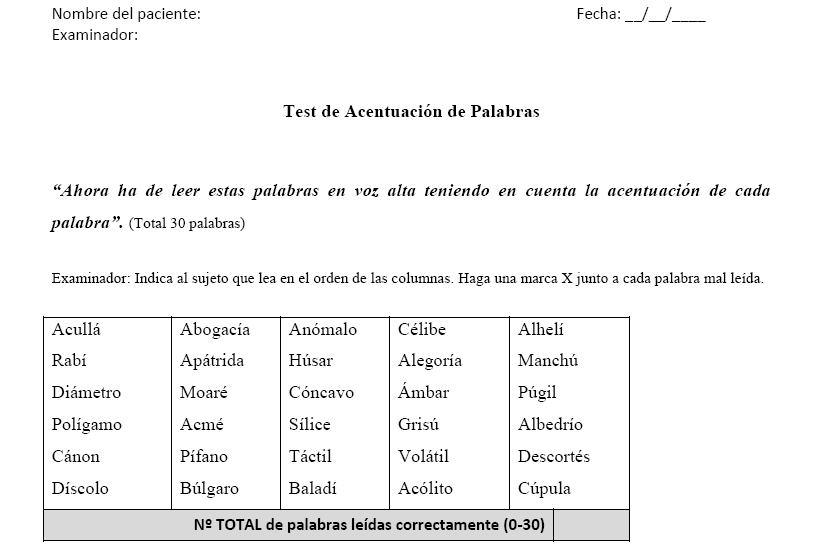

Supplement: FIGURE S1 — Instructions and correction of the WAT in Spanish for the rater’s use. [file Image_1.TIF]

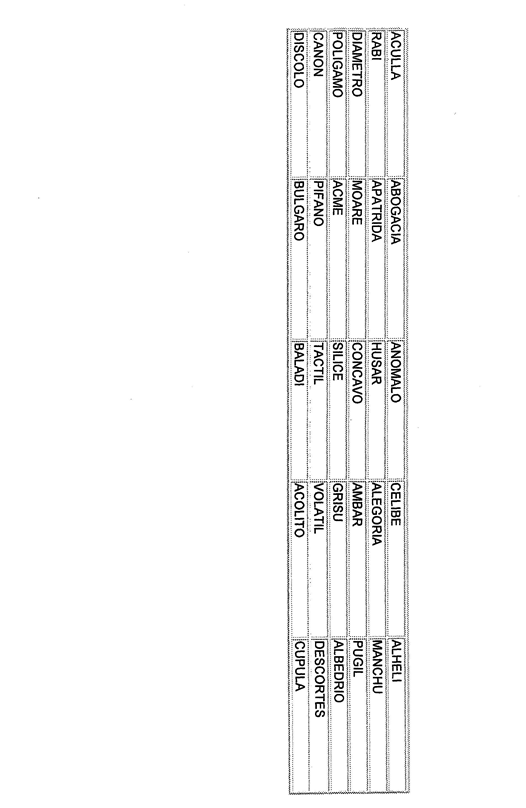

Supplement: FIGURE S2 — Stimulus sheet of the WAT. [file Image_2.TIF]
